# The politics of multilateral environmental agreements lessons from 20 years of *INEA*

**DOI:** 10.1007/s10784-022-09567-6

**Published:** 2022-02-23

**Authors:** Agni Kalfagianni, Oran R. Young

**Affiliations:** 1 Copernicus Institute of Sustainable Development, Utrecht University, Utrecht, Netherlands; 2 Bren School of Environmental Science and Management, University of California Santa Barbara, Santa Barbara, USA

**Keywords:** Effectiveness, Implementation, Institutional design, Institutional politics, Lessons learned, Multilateral environmental agreements

## Abstract

This review article addresses the question: What lessons can we learn from work published in *International Environmental Agreements: Politics, Law and Economics* regarding the politics of multilateral environmental agreements? What are the implications of these lessons for those responsible for creating and administering these agreements? Based on an analysis of 147 articles published over the past 20 years, the article explores issues of institutional design, institutional politics, implementation, and effectiveness. It concludes that key conditions for success in this realm include: (a) developing a toolkit that is not limited to rules-based governance, (b) paying attention to matters of implementation, (c) bearing in mind the overall regime complex, (d) developing effective leadership based on credibility and accountability, and (e) allowing for institutional adaptation.

## A focus on politics

This article, one of a collection of papers assessing the contributions of *International Environmental Agreements (INEA)* over a span of 20 years*,* directs attention to political matters in contrast to economic and legal issues. The boundaries separating these concerns are by no means clearcut. This explains the tight links reflected in the concept of political economy and in many accounts of law and politics, even among those who work in universities where there are explicit distinctions among departments of politics, economics, and law. In other settings where these distinctions are blurred both in practice and in theory, directing attention to matters that are predominantly political becomes even more challenging. Nevertheless, in reviewing articles published in *INEA* from a political perspective, we seek to highlight the nature of the processes through which multiple actors arrive at and implement collective choices regarding the establishment of environmental governance systems, the efforts of individual actors and coalitions to exercise influence in the creation and administration of such arrangements, and the results flowing from the operation of these governance systems.

In conducting this assessment, we have focused on multilateral environmental agreements (MEAs). The future of multilateralism—i.e. coordination among three or more states in pursuing common goals—is heavily debated. Over the past twenty years, the world map has been redrawn in terms of changing geopolitics, trade patterns, human mobility, and income levels (Burch et al., 2019). Emerging economies, especially China, have become major economic players in the global arena; roles are being reshaped within blocs driven, for example, by new development banks, especially in Asia. Simultaneously, the European Union has experienced for the first time a withdrawal from its membership with the UK officially leaving on 31 January 2020. The lessons we draw from this analysis can be important stepping stones for more successful multilateral cooperation in the future.

We pay particular attention to the findings presented in some 147 articles the editors of the journal identified for their relevance to these political concerns. Focusing on these articles limits our analysis. Nonetheless, by keeping our review as broad as possible, we seek to cover *INEA’s* contribution to this field. Reviewing the content of these articles inductively, we have concluded that it makes sense to group their contributions into four clusters of issues relating to (i) matters of institutional design including the selection of key features included in MEAs established to deal with specific issues, (ii) efforts on the part of actors to exercise influence over the creation and adjustment of such agreements, (iii) processes involved in the implementation of the provisions of agreements once they are put in place, and (iv) factors determining the results flowing from the operation of MEAs. The next four sections take up these clusters. We then consider opportunities for growth, identifying some themes that have not yet achieved prominence in articles published in *INEA* but that offer promising avenues for contributions in the coming years. In a brief concluding section, we highlight a number of lessons arising from our assessment that may prove helpful to those seeking to address needs for governance at Stockholm + 50 and similar settings.

## Matters of institutional design

We know, of course, that both the creation and the implementation of MEAs feature political processes. In most cases, regime formation (Krasner, 1983) involves institutional bargaining in which multiple actors with overlapping but far from identical interests engage in efforts to hammer out the terms of mutually acceptable agreements (Young, 1994). The contents of the resultant agreements generally reflect compromises needed to arrive at assemblages of provisions that are acceptable to all major participants or negotiating blocs. The requirements of making progress in the course of institutional bargaining typically take precedence over any commitment to the adoption of arrangements conforming to coherent institutional designs. The interplay of divergent interests does not come to an end once the provisions of an MEA are in place. Both member states and influential non-state actors continue to pursue their own interests in the course of moving an agreement from paper to practice and administering it on a continuing basis. That is one important reason why what analysts call rules in use in environmental governance often differ significantly from the rules on paper (Ostrom, 1990).

Nevertheless, these realities have not dampened the growth of interest in institutional design. We seek to devise MEAs in which the key components of these arrangements are well matched with the major features of the problems they are intended to solve. This means going beyond panaceas, setting aside formulaic prescriptions to concentrate on creating institutional arrangements tailored to the characteristics of the problem at hand. The result is an interest in what analysts have described as institutional diagnostics (Ostrom, 2007; Young, 2002). Pursuing this agenda may not produce general propositions applicable to large universes of cases. But if done well, a focus on design holds out the prospect of generating insights that will prove useful to those responsible for creating regimes intended to solve specific environmental problems.

Starting at the lowest administrative level, we can consider design issues pertaining to specific components of individual MEAs. We can then proceed step-by-step to ask questions about the design of regimes in holistic terms, issues pertaining to clusters of regimes or what analysts treat as regime complexes—i.e. “the array of partially overlapping and nonhierarchical institutions governing a particular issue-area” (Raustiala & Victor, 2004: 278–279)—and finally concerns relating to interactions between or among regimes dealing with matters arising in distinct issue domains. Reviewing the contributions to *INEA* over the last 20 years*,* it seems clear that many of those interested in matters of design have directed attention to the lowest administrative level on this scale. But increasingly, we are coming to recognize the importance of issues of institutional design arising at higher levels (Ivanova, 2007; Lejano, 2006).

A focus on regime components raises questions regarding the design of steering mechanisms, decision-making procedures, organizational arrangements, monitoring and verification systems, compliance mechanisms, and amendment procedures (Kemp, 2016). There is a lively interest in the design of suitable policy instruments, including debates about the relative merits of permit systems and carbon taxes as instruments for controlling emissions of greenhouse gases and the conditions under which individual transferable quotas (ITQs) make sense in avoiding stock depletions and achieving efficiency in marine fisheries (Anger et al., 2016; Boockman and Thurner 2006; Cole, 2012; Dooley & Kartha, 2018; Haddad & Palmisano, 2001; Hovi & Holtsmark, 2006; Talberg et al., 2018;).

Similar comments are in order regarding the role of organizational arrangements needed to administer regimes and compliance mechanisms. For instance, analysts have considered the pros and cons of locating the secretariats for individual MEAs under the umbrella of the UN Environment Programme (UNEP). They have also considered the extent to which regime members respond to the logic of consequences or the logic of appropriateness in considering the design of compliance mechanisms (Hovi & Holtsmark, 2006; Kallbekken and Hovi 2006; Tveit, 2018). For regimes designed to tackle problems on a step-by-step basis, similar concerns arise regarding the development of procedures for strengthening requirements over time. For example, analysts have examined the relative merits of the global stocktake procedure built into the 2015 Paris Climate Agreement and the procedures included in the Montreal Protocol regarding the acceleration of phaseout schedules and the addition of new families of chemicals to the set of substances regulated under this MEA (Gareau, 2010; Milkoreit & Haapala, 2019).

Scaling up to a holistic perspective on regimes, a different set of design considerations comes into focus (Hourcade & Shukla, 2015; Kameyama, 2004; Kanie et al., 2010; Verbruggen, 2011). A fundamental issue here concerns the nature of the mechanisms regimes employ to steer the behaviour of their members and those subject to their jurisdiction (Michaelowa et al. 2005). The bulk of the contributions to *INEA* adopt a rules-based or regulatory approach in which the key to steering is the adoption of requirements and prohibitions applicable to the behaviour of specific groups of subjects (e.g. emitters of greenhouse gases). But it is now clear that there are alternatives to rules-based governance in which other mechanisms come into play in efforts to steer behaviour (Bromley, 2001; Morseletto et al., 2017; Young, 2001). Recent analyses of the UN Sustainable Development Goals, for example, have drawn attention to goals-based governance in which those responsible for regime design seek to galvanize the behaviour of actors in pursuit of common goals over a specified period of time (Chasek & Wagner, 2016; Gellers, 2016; Gupta, 2002; Gupta & Lebel, 2020; Gupta & Vegelin, 2016). Governance through goals differs from rules-based governance in a number of ways, including the inclusive goal-setting process, the non-binding nature of the goals, the reliance on weak institutional arrangements, and the extensive leeway that states enjoy (Biermann et al., 2017). Behavioural mechanisms are not the only focus of attention for those who approach institutional design at this level. For instance, analysts have considered tradeoffs between legally binding arrangements whose provisions are relatively weak and stronger arrangements articulated in more informal agreements that do not get bogged down in the political processes surrounding ratification (Andresen et al., 2013; Böhmelt & Butkute, 2018; Yamagata et al., 2017).

Design questions relating to regime complexes have garnered increased attention in recent years as we recognize that many issue domains (e.g. climate, plant genetic resources, Antarctica) feature institutional elements that interact with one another but are not organized into hierarchical structures. This draws attention to the importance of managing institutional interplay to avoid interference among the elements of regime complexes and to promote synergy so that the whole evolves into a governance system that is greater than the sum of the parts (Oh & Matsuoka, 2017; Overland & Reischl, 2018; Sugiyama & Sinton, 2005; Wilson, 2008). Some responses focus on efforts to endow collections of elements with common cognitive structures (e.g. ecosystem-based management) or normative commitments (e.g. the precautionary principle). Analysts have explored ways to create administrative arrangements that can function as orchestrators of institutional interplay to minimize fragmentation (Abbott et al., 2020). Some have gone a step further to suggest that it would be helpful to provide MEAs dealing with related concerns (e.g. the Basel Convention on the Control of Transboundary Movements of Hazardous Wastes and Their Disposal, the Rotterdam Convention on the Prior Informed Consent Procedure for Certain Hazardous Chemicals and Pesticides in International Trade, and the Stockholm Convention on Persistent Organic Pollutants) with common administrative arrangements to maximize the prospect that they will perform in a mutually supportive manner.

Moving up again, we come to issues arising when regimes operating in different issue domains interact with one another (e.g. interactions between various MEAs and the global regime governing trade) (Hourcade et al., 2015; Jones, 2002). Some have noted problems arising from the use of the World Trade Organisation’s (WTO) dispute settlement mechanism to handle disputes about environmental issues (e.g. the tuna-dolphin controversy). These concerns have produced proposals for the creation of a separate dispute settlement mechanism more attuned to the requirements of environmental protection. New concerns of this sort have come into focus in recent years. For instance, many have noted that climate change is a critical factor affecting the success of efforts to protect biological diversity under the terms of the Convention on Biological Diversity. But there is no mechanism for addressing interactions between the biodiversity regime and the climate regime. Similar concerns have come into focus in connection with efforts to promote progress toward the achievement of the UN’s Sustainable Development Goals. While some see each goal as a distinct issue domain, others note that many of them are tightly coupled. This suggests a need to consider the role of nexuses such as the energy/water/food nexus as well as the observation that there are fundamental links between the goals dealing with individual welfare like poverty, health, and education and the goals dealing with systemic concerns like the Earth’s climate system and the world’s oceans (Boas et al., 2016). These are early days in thinking about issues of institutional design arising from these linkages across issue domains and whether or not a separate dispute settlement mechanism is necessary to address challenges between regimes. But those interested in matters of institutional design will need to devote much more attention to these matters going forward.

## Institutional politics

Institutional politics encompasses the political dynamics among actors seeking to exercise influence in international environmental governance. MEAs are created and adapted by states and, to a lesser extent, non-state and subnational actors, such as cities. Much of the discussion emerging among contributors to *INEA* brings highlights distinctions between small and big states, developed and developing countries, and participants from the North and South in efforts to influence the course of institutional politics.

Many analysts focus on the role of leadership. There is considerable interest in the leadership roles of small European countries, such as the Netherlands (Anderson and Mol 2002), Norway (Rosendal, 2007), and Switzerland (Schultz et al. 2016). These studies emphasize the role of intellectual leadership and scientific expertise as well as the tactical skills of national delegations, particularly in the absence of structural economic power and size. This is often contrasted with the role of the USA analysed through the prisms of unilateralism (Andresen et al., 2013) and market environmentalism (Boyd et al., 2008), frequently producing impediments to agreeing on common goals.

The leadership of the European Union (EU) has attracted attention as well (Afionis & Stringer, 2014; Parker & Karlsson, 2017; Vogler and Stephan 2007). Analysts note the normative power of the EU in this context but also raise concerns about its leadership being perceived as a form of soft imperialism by other states, particularly in the Global South. The tensions between internal policy coherence and the EU’s aspiration to exercise global environmental leadership are another focus (Afionis & Stringer, 2014). Here studies point out that credibility is essential to enhance persuasion. They argue that for the EU to be able to convince others to follow its lead, it needs to live up to its own high standards of environmental protection (Vogler and Stephan 2007). The competition for leadership in a fragmented governance landscape is also analysed, particularly as far as the EU’s competition with states like the USA and China is concerned, revealing the impacts of different visions and institutional design preferences for environmental governance (Parker & Karlsson, 2017).

Contributors also explore the roles international organizations, such as UNEP, play in the political dynamics of MEAs. To illustrate the case, some contributors (Andresen et al., 2013) seek to explain why negotiations aimed at producing a legally binding mercury convention got underway despite opposition from the USA and key emerging economies, especially China and India which preferred a voluntary approach instead. Especially for the USA, a voluntary approach was consistent with the ideological stance of the Bush administration on international environmental issues and was supported on the basis of its supposedly superior effectiveness in getting things done on the ground (ibid.). The analysis by Andresen et al. (2013) demonstrates the importance of intellectual leadership in changing the position of powerful states, where a leader is regarded as someone who “produces intellectual or generative systems of thought that shape the perceptions of those who participate in institutional bargaining” (Young, 1991: 300). Such leadership involves knowledge creation and learning rather than coercion. In the case of mercury, UNEP provided knowledge-based leadership during the negotiations, providing new information not so much on the seriousness of the problem as on other factors that induced many states to rethink their interests regarding the trade dimension. For knowledge-based leadership to succeed, however, it must be combined with structural or power-based leadership (e.g. American leadership following a change in domestic politics) and interest-based leadership (e.g. the proactive efforts of the EU and others who were proponents from the start).

Contributors to *INEA* also note the leadership of the conference presidency in promoting successful negotiations. The Mexican presidency during the 16th Conference of the Parties (COP) of the United Nations Framework Convention on Climate Change, for example, was key in crafting the 2010 Cancun Agreements (Park, 2016). This provided a sharp contrast with the lackluster leadership of the Danish government widely regarded as a source of the failure of COP 15 in 2009. Specifically, analysts point to factors like the control of the agenda, process management through transparency and engagement in handling subgroup meetings, the production of a single negotiating text in a timely fashion, and more generally the cultivation of trust among negotiators.

Less attention is paid to laggards. Djoundourian argues, for instance, that the Arab world, which is considered a laggard in climate change policies, is shifting toward making commitments through investments in environmental infrastructure and developing a variety of mitigation and adaptation projects (Djoundourian, 2021). Research also highlights that countries can be leaders or laggards depending on the complexities of national politics (Arnoldussen, 2019). One example is the UK which operates as laggard in emissions standards but as leader in adoption of integrated pollution control (Héritier et al. 1996 in Arnoldussen, 2019).

Analyses of institutional politics also point to the role of cooperative blocs, particularly among developing countries, in exerting influence. The possibilities of collaboration among developing countries through mechanisms like the G77 are substantial, despite significant differences in prosperity and responsibility for environmental problems (e.g. levels of greenhouse gas emissions) (Kasa et al., 2008). The limited agency of African countries in the creation of international environmental agreements, particularly on climate change, is notable. These countries not only lack the capacity to send large delegations to negotiating sessions; they also are unable to find a common voice regarding major issues (Atela et al., 2017).

Numerous studies focus on the BASIC countries (Brazil, South Africa, India, and China) and especially China in institutional politics. Some underline these countries taking an active interest in international environmental governance when environmental concerns (e.g. reducing greenhouse gas emissions) are coupled with “development” to deliver benefits and reduce conflicts (Shukla & Dhar, 2011) and when linking environmental issues to financial assistance and technology transfer (Walsh et al., 2011). A key concern has been the commitment of these countries to a post-Kyoto world, particularly in light of the emergence of regional initiatives such as the Asia Pacific Partnership (APP) focusing on the reduction of energy intensity in growth strategies (Heggelund & Buan, 2009). Some also fear that emerging regional initiatives like the East Asia Low Carbon Growth Partnership initiated by Japan will lead to more fragmented environmental governance on a global scale (Oh & Matsuoka, 2017).

Few articles in *INEA* have focused on the role of non-state and subnational actors in MEAs. But this is expected to change as national commitments are considered insufficient to limit temperature increases to 2 °C, much less 1.5 °C. Research highlights the importance of orchestration, treated as the alignment between “orchestrators” (e.g. international organizations and governments) and “intermediaries” (e.g. city networks and partnerships) in inspiring greater ambition in achieving environmental objectives (Chan et al., 2018). The advice emerging from these studies is for orchestrators not only to pay attention to large-scale impacts but also to engage in small-scale experiments where local actors may prove important for long-term transformation and change.

## Processes of implementation

Implementation encompasses the process of putting MEAs into effect. In the past, analysts often distinguished between the creation of governance systems, which they saw as a political process, and the administration of the resultant arrangements, which they regarded as a matter of public administration. On this account, administration was the concern of dedicated professionals who would pursue the goals embedded in MEAs in the absence of political biases. Now, we understand this is a false dichotomy (Pressman & Wildavsky, 1984). Those pursuing various interests do not switch gears once they accept the terms of an agreement, shifting from advocacy to good-faith efforts to implement the provisions of MEAs. Both member states and others continue to pursue their objectives during the processes involved in moving agreements from paper to practice. This is one reason why we often observe a sizeable gap between the ideal and the actual in comparing the results flowing from the operation of governance systems with what might be expected from an examination of the provisions of the relevant agreements. As a result, it is not surprising that there has been a substantial growth of interest in the politics of implementing MEAs during the lifetime of *INEA*.

Implementation takes place at two distinct levels: within administrative arrangements operating at the international level and within administrative arrangements operating within the governments of member states. Most MEAs are lightly administered at the international level. They feature periodic sessions of conferences (or meetings) of the parties and relatively modest secretariats; they often depend on outside bodies for the provision of scientific advice and for rendering authoritative interpretations to settle disputes. Nevertheless, analysts have observed a trend over time toward the growth of more substantial international administrative arrangements. Domestically, governments generally assign responsibility for the administration of national participation in MEAs to designated agencies, such as the Environmental Protection Agency (EPA) in the USA. Whether these agencies receive adequate material support to play their roles effectively or exhibit strong leadership in moving the terms of agreements from paper to practice varies from case to case.

Analyses at the international level have focused on the operation of conferences (meetings) of the parties treated as executive bodies responsible for making decisions (e.g. annual decisions setting quotas in fisheries), overseeing programmatic activities, and assessing needs to strengthen regimes (Dessai et al., 2005). Recently, interest in the roles of secretariats along with various committees and working groups has risen. These bodies generally lack authority and are severely constrained with regard to the availability of material resources. Yet in many situations, they are able to make a difference through the exercise of soft power (Jinnah, 2014). They can identify emerging issues. They can nudge key actors to take the initiative by making recommendations and then following up to publicize whether or not parties act on these recommendations. In some cases, heads of secretariats are able to exercise intellectual or entrepreneurial leadership regarding the implementation of MEAs.

Another key issue regarding implementation concerns the extent to which actors comply with the terms of international regimes (Carbonell, 2016; Hovi & Holtsmark, 2006; Kallbekkan and Hovi 2007). Traditional thinking in this area emphasized the importance of enforcement mechanisms and drew attention to the difficulties of devising effective approaches to enforcement in international settings. Recent work on compliance, however, explores different sources of compliance (Tveit, 2018). For example, compliance rises when stakeholders feel they have had an opportunity to participate in shaping the provisions of regimes and regard the results as legitimate. There is a relationship between compliance and the extent to which those subject to a regime’s prescriptions feel the provisions of the regime meet standards of equity or fairness. In thinking about compliance at the international level, we need to adopt an approach that goes beyond utilitarian calculations aimed at maximizing compliance while minimizing the material resources devoted to achieving compliance.

We are also witnessing a growing interest in issues involving implementation extending beyond individual MEAs. One question concerns clustering (Oberthür, 2002). Some have suggested that grouping regimes dealing with related issues (e.g. atmospheric issues or marine issues) would support efforts to move these arrangements from paper to practice. An even broader issue concerns the extent to which it would be helpful to upgrade UNEP into a UN Environment Organization or to create a World Environment Organization as a counterpart to the World Trade Organization (Biermann, 2002). An inconclusive debate has arisen regarding the extent to which bringing a large number of MEAs into alignment under the auspices of such an overarching body would prove helpful from the perspective of implementation (Oberthür & Gehring, 2004). What does seem clear, however, is that moving toward structural adjustments is easier said than done (Vijge, 2013). Repeated efforts to strengthen UNEP, for instance, have produced limited results for years (Rosendal, 2007), although UNEP was strengthened in 2012 with the adoption of a resolution at the 67th session of the UN General Assembly that granted UNEP universal membership and called for increased resources.

Regarding the implementation of the provisions of international regimes within the political systems of member states, the first thing to note is great variation among the members of MEAs in terms of political operating systems, socioeconomic development, and national culture (Carbonell & Allison, 2015; Gray, 2003; Najam, 2005; Schreurs, 2005; Tacconi et al., 2008; Zaharchenko & Goldenman, 2004). Consider the contrast between China and the USA. In China, the critical factors involve endorsement on the part of the leadership of the Chinese Communist Party and subsequent prioritization in the provisions of Five-Year Plans. In the USA, by contrast, the challenge is to achieve ratification on the part of the Senate followed by the passage of implementing legislation. Short of this, the fate of MEAs is determined by shifting executive preferences as exemplified by the treatment of the 2015 Paris Climate Agreement on the part of the Obama, Trump, and Biden Administrations (Skodvin & Andresen, 2009). These variations explain the popularity of the idea of Nationally Determined Contributions (NDCs) incorporated in the 2015 climate agreement. NDCs allow individual regime members to tailor their commitments to domestic circumstances. In principle, these commitments are subject to periodic strengthening through procedures like the climate agreement’s global stocktake (Milkoreit & Haapala, 2019).

Analysts have examined a range of factors deemed relevant to the implementation of the provisions of MEAs within the political systems of member states (Andresen and Butenschoøn 2001; Böhmelt & Butkute, 2018; Buchner & Carraro, 2006; Heggelund & Backer, 2007; Lebel et al., 2018; Poussenkova, 2003; Salmi, 2008). Measures that immunize international commitments from the ups and downs of domestic politics make a difference (Law & Kriwoken, 2017). One explanation for the sharp contrast regarding implementation of the Montreal Protocol and the UN Framework Convention on Climate Change in the USA, for example, emphasizes that procedures for implementing the ozone regime are embedded in the Clean Air Act Amendments of 1990 passed by large bipartisan majorities in both houses of Congress. Another factor is the dedication of those in administrative agencies who assume responsibility for the implementation of the provisions of regimes (Zhou & Mori, 2011). Line agencies often become champions of the regimes they administer (Dai, 2005). Except in cases where an issue becomes a focus of partisan politics (e.g. climate change), dedication on the part of responsible agencies may prove sufficient to ensure that key requirements are met on a routine basis. The introduction of procedures to assist member states to fulfil obligations under the terms of MEAs also emerges as an important determinant of success (Smits et al., 2014). For example, the Montreal Protocol Multilateral Fund has strengthened the capacity of developing countries to avoid reliance on ozone-depleting substances. While implementation at the domestic level is clearly a political process, there are numerous factors beyond the mainstream of partisan politics that can help us understand striking differences in the fate of specific MEAs in the domestic arenas of member states.

## Determinants of effectiveness

Contributors to *INEA* have been grappling with how to assess the effectiveness of MEAs. We need to recognize at the outset differences in how analysts define effectiveness. Some define effectiveness in terms of the ability to solve the problems prompting the establishment of MEAs (Andresen & Hay, 2005; Young, 1999); others refer to outputs, outcomes and impacts (Andresen, 2007), and still others resort to even broader concepts like success (Schreurs, 2005; Zhou & Mori, 2011).

Drawing on Miles et al. (2002), some analysts pay attention to problem characteristics, which can be benign or malign depending on the configuration of actor interests a problem generates. A problem is benign when preferences are identical and becomes malign the further away we move from this harmony. Some characterize the ozone problem as benign for the European Community which explains why the EU and member states have reached targets effectively in a short time (Naes, 2003). In contrast, others highlight the malignity of chloride issues in international water negotiations making compromises between upstream and downstream states difficult (Dieperink, 2011). 

Some determinants of effectiveness lie in the design of agreements. Analysts have emphasized the differentiation of obligations among member states based on different interests, since differentiation is expected to align national interests with the common interest (Andresen & Hay, 2005). Others suggest building common knowledge and making use of external factors, such as exogenous shocks can enhance effectiveness. Another design feature that matters is finding a balance between too much flexibility in compliance mechanisms and too little flexibility (Boockman and Thurner 2006). Some regard making it difficult to amend treaties as a key to effectiveness (Boockman and Thurner 2006). But MEAs that are too difficult to amend are likely to fail when key features of the problem change rapidly.

Time emerges as another important factor. The time required to negotiate an agreement matters because certain issues relevant at the outset may lose their urgency if negotiations drag on for too long (Boyd et al., 2008). The time interval involved in assessments of effectiveness also matters (Grunding, 2012). Suggesting amendments to the Oslo-Potsdam approach to measuring effectiveness (Hovi et al., 2003), several scholars have argued that effectiveness needs to be assessed over a specified time interval (Grunding, 2012). This requires analysts to make careful choices regarding beginning and end points, including considering the time prior to the date of entry into force.

The issue of scale also matters. Making an agreement that does not match the capacity of actors to implement it at the national or local level, for example, reduces the effectiveness of the agreement (Boyd et al., 2008). Similarly, there are links between joint commitments and domestic policy ambitions (Skaerseth, 2003). The depth of domestic ambitions required depends on the type of commitments, issue-area and actors involved. Avoiding the creation of hegemonic regimes is important for the domestic effectiveness of policies (Hussein & Grandi, 2017). Strong domestic scrutiny and support is crucial for the effectiveness of international environmental treaties as the case of Tasmania’s protection of tall-eucalyptus forests attests (Law & Kriwoken, 2017).

The domestic political systems of member states also matter for effectiveness. Evidence demonstrates an overall positive relationship between democracy and state commitment and compliance with international environmental agreements (Carbonell & Allison, 2015). However, this is not always the case, as the contrast between the recent policies of China and the USA regarding climate change suggests. One study of interactions between access to clean water and government type concludes that democratic governments experiencing limited access to clean water are less likely to comply with international environmental agreements. The opposite is the case for authoritarian governments (Carbonell & Allison, 2015). This suggests that authoritarian states are more likely to seek international cooperation than democratic states, at least in the case of water scarcity. Questions of leadership and power appear but less often in relation to institutional politics as discussed earlier. Some authors highlight the role of power in hegemonic contexts where the “first among equals” has the power to determine choices other states make particularly in non-violent conflicts (Zeitoun et al., 2010). Others emphasize the hegemon’s vulnerabilities and how these can be exploited by others to achieve desirable outcomes (Petersen-Perlman & Fischhendler, 2018).

Contributors also consider the role of learning and knowledge transfer as sources of effectiveness. Learning occurs through interpersonal communication and reflection that leads to the recognition of shared values as well as through the recruitment of new administrative personnel (Haas, 2001). Knowledge sharing is important since even powerful actors are limited by their understanding of the institutional options available (Young 2004). Analysts have identified three mechanisms through which institutions shape the growth of knowledge: (1) structuring research agendas through framing, and therefore prioritizing certain issues; (2) privileging certain types of knowledge claims affecting how key issues are understood, and (3) guiding the application of knowledge to specific policy concerns enhancing the credibility of certain streams of research. Examples from climate, biodiversity and fisheries illustrate that while the influence of all three mechanisms is subject to doubt in specific cases, they have created knowledge in several issue areas proving vital for future effectiveness.

Similarly, analysts have attributed the success of the climate negotiations in Paris in 2015 versus the failure in Copenhagen in 2009 to efforts to draw lessons from past failures (Rietig, 2014). They highlight the relevance of workshops and roundtables where country representatives showcase their mitigation plans and low carbon development initiatives, contributing to raising ambitions and creating group pressure in other countries. Scale also matters. Knowledge sharing within the UNFCCC and between the domestic level and international negotiations made climate negotiations less confrontational and emphasized identifying co-benefits rather than who should reduce emissions. In this way, the institutionalization of knowledge sharing within the UNFCCC becomes a basis for more ambitious climate policy in the future.

Others take a broader perspective emphasizing system-level indicators where the evaluation of effectiveness is less a matter of how MEAs affect problem-solving and behavioural changes than how they affect the system. Studies on reducing fragmentation and enhancing the coherence of MEA systems through clustering offer an example (Oberthür, 2002). Clustering involves “grouping a number of international environmental regimes together so as to make them more efficient and effective” (Moltke 2002: 3). Clustering can occur both in organizational elements, such as secretariats, and in MEA functions, including responses to non-compliance, implementation review, or financial assistance. But there is a need for caution about the potential for clustering to lead to improving environmental governance (Oberthür, 2002). Clustering needs to be approached on a case-by-case basis.

A related concept emphasizes interplay arising from multifaceted interactions among institutions (Young, 1999). One study finds this concept useful in analysing shorebird conservation (Wilson, 2008). The concept of interplay, along with fit and scale, has been used to analyse the roles institutions play in both causing and confronting environmental problems by the international research project on the Institutional Dimensions of Global Environmental Change through empirical studies of marine, terrestrial, and atmospheric systems (Young, 2003). More recently, analysts have introduced the concept of orchestration to examine a particular facet of institutional interplay (Abbott, 2020). An analysis of 40 transnational initiatives involving climate change adaptation suggests that leadership and orchestration are central in achieving behavioural changes on the part of target groups (Dzebo, 2019). Achieving orchestration cannot rely solely on a single powerful orchestrator. Rather, good process management on the part of an independent secretariat with full-time staff and clear decision-making structures, funding and capacity to orchestrate as well as a high level of institutionalization through binding rules for partners and good coordination with international regimes are keys to successful orchestration. The role of orchestration as a determinant of effectiveness at the domestic level is also noted. For example, inter-agency coordination mechanisms designed to adjust the roles of various governmental agencies in addressing environmental problems have contributed to effectiveness in some cases (Zhou & Mori, 2011).

## Opportunities for growth

In the years since the founding of *INEA,* major environmental problems like climate change and the loss of biological diversity have become more severe and increasingly complex. Today, they are intertwined with socioeconomic inequalities, political polarization, and the COVID-19 pandemic. Forward-looking research on the politics of MEAs must consider these developments in addition to the familiar concerns regarding the design, creation, and performance of agreements aimed at alleviating specific problems. To round out our assessment, we identify several themes reflecting relevant trends in the work of contributors to *INEA* and providing opportunities for innovative research.

Most studies of MEAs adopt a regulatory or rules-based approach to governance. But there are other behavioural mechanisms that come into play in addressing specific needs for governance. Recent articles in *INEA* and elsewhere dealing with the UN’s Sustainable Development Goals have drawn attention to arrangements highlighting goals-based governance. Additional options worth considering include the creation of arrangements featuring principles-based governance, pledge-based governance, and standards-based governance (Young, 2021: Ch. 3). One result of expanding our thinking about behavioural mechanisms is an interest in hybrid arrangements. The 2015 Paris Climate Agreement, for example, combines a well-defined goal with a pledge-and-review system featuring Nationally Determined Contributions, and a number of regulatory measures dealing with monitoring, reporting and verification. Although the jury is out on the long run success of the Paris Agreement, the key lesson here is that there are opportunities for enriching our toolkit in coming to terms with complex needs for governance.

We note also the movement from a focus on single MEAs to an interest in regime complexes. Going forward, we need to think about institutional interplay across issue domains (e.g. trade and the environment, health and the environment) and about interactions between public and private institutions. This concern animates the emerging interests in clustering, interplay, and orchestration. Beyond this lies a consideration of potential synergies between MEAs and the expanding universe of private governance arrangements. A topic of particular interest encompasses the conditions under which public–private partnerships can contribute to addressing needs for governance in ways that not only alleviate particular problems but also enhance values like legitimacy and accountability (Andonova, 2017).

The relative lack of attention both within the research community and in the realm of policy devoted to the circumstances of the poor is another concern. Questions regarding justice are surfacing with increasing intensity, including calls for justice for future generations and for non-human species (Celermajer et al., 2020). Some now propose a broader reconceptualization of justice based on the idea of planetary justice (Biermann & Kalfagianni, 2020). Prioritizing the concerns of poor peoples may be crucial to ensuring planetary integrity as well as promoting basic human rights (Kashwan et al., 2020). Future research needs to engage more closely with the difficult ethical and political questions of global reallocation of wealth, including ways to enhance the influence of the poor at all levels.

Finally, there is a need to avoid assuming that the global order will continue indefinitely to take the form of an international society in the sense of a society composed predominantly, if not exclusively, of sovereign states. Interest will continue in the creation and administration of MEAs in the form of international legally binding instruments. But we should pay more attention to the role of non-state actors including transnational corporations and civil society organizations and to the future of various types of partnerships. This suggests an enquiry into the debate between those who argue achieving sustainability will require an expanded role of the state in restructuring social institutions as well as redistributing income and wealth (Eckersley, 2021) and those who advocate shifting away from nation states and embracing a variety of non-state actors in pursuit of global cooperation (Mert, 2020). This debate will intensify as we seek to understand the sources of past failures and to envision new approaches to coexistence, cohabitation, and codevelopment. *INEA* is well-placed to provide a forum for the discussion of issues of this type.

## Lessons learned

It would be naive to adopt a cookbook approach to the creation and implementation of MEAs. Every governance system addresses a unique problem and operates within a particular constellation of economic, political and social forces. Experienced negotiators and administrators are acutely aware of this reality. Nevertheless, the members of the research community including those who have contributed to *INEA* have developed a number of insights about institutional politics that may be helpful to those seeking to come to terms with newly emerging needs for governance. Here*,* we single out five lessons relating to the politics of MEAs that strike us as particularly relevant to the concerns of practitioners.

### Employ a toolkit not limited to rules-based arrangements

All successful governance systems use behavioural mechanisms to steer the actions of those subject to their provisions. Sometimes regulations featuring requirements and prohibitions work well. But different mechanisms produce better results in other situations. It makes sense to develop a well-stocked toolkit and to devote attention to selecting the best behavioural mechanism or combination of mechanisms to achieve success in responding to specific needs for governance.

### Pay attention to matters of implementation in addition to issues of design

The process of implementing governance systems is just as political as the process of negotiating the provisions of MEAs. Arrangements that seem attractive on paper may run into major problems at the stage of implementation. What looks like a second-best response to a need for governance on paper may prove more effective in practice than an alternative that seems preferable in principle.

### Bear in mind governance systems never operate in a vacuum

As the number of MEAs grows, the density of arrangements operating in any given issue domain increases. Institutional interplay can lead to interference, but it can also generate synergies. To maximize the effectiveness of individual governance systems, it is essential to pay attention to the dynamics of what analysts call regime complexes.

### Leaders matter as much as laggards

Leadership is important in meeting needs for governance; it can come from different actors operating at different levels of social organization. Laggards are equally important. Understanding the circumstances under which laggards can change is crucial for efforts to achieve sustainable outcomes.

### Develop institutional reflexivity

While MEAs are designed to operate in specific settings, they also need to be responsive to changing circumstances. Rigidity leads to failure when rapid changes in key features of the problem or the broader setting change are not recognized. Effectiveness requires mechanisms to adapt to changes without undermining efforts to pursue priority goals.

## Abbreviations

APP: Asia pacific partnership
BASIC countries: Brazil, South Africa, India, and China
CDM: Clean development mechanism
COP: Conference of parties
EPA: Environmental protection agency
EU: European union
G77: Group of 77
IDGEC: Institutional dimensions of global environmental change
INEA: International environmental agreements: politics, law and economics
ITQs: Individual transferable quotas
LDCs: Least developed countries
MEAs: Multilateral environmental agreements
NOAA: National oceanic and atmospheric administration
NDCs: Nationally determined contributions
UNEP: United nations environment programme
UNESCO: United nations educational, scientific and cultural organization
UNFCCC: UN framework convention on climate change
WTO: World trade organisation
